# Identification of sepsis-associated encephalopathy risk factors in elderly patients: a retrospective observational cohort study

**DOI:** 10.55730/1300-0144.5491

**Published:** 2022-07-09

**Authors:** Guangyong JIN, Shengyun WANG, Jiayi CHEN, Wei HU, Ying ZHU, Shaosong XI

**Affiliations:** 1 Department of Critical Care Medicine, Affiliated Hangzhou First People’s Hospital,Zhejiang University School of Medicine, Hangzhou, Zhejiang, P.R. China; 2 Department of Emergency and Critical Care Medicine, Changzheng Hospital. Second Military Medical University, Shanghai, P.R. China

**Keywords:** Sepsis-associated encephalopathy, sequential organ failure assessment scores, acute physiology and chronic health evaluation

## Abstract

**Background/aim:**

Sepsis-associated encephalopathy (SAE) is a severe complication of sepsis that affects upwards of half of all sepsis patients. Few studies have examined the etiology and risk factors of SAE among elderly patients. This study was designed to explore the epidemiology of SAE and the risk factors associated with its development in elderly populations.

**Materials and methods:**

This was a retrospective analysis of elderly sepsis patients admitted to our intensive care unit between January 2017 and January 2022. We then compared non-SAE and SAE groups concerning baseline clinicopathological findings, underlying diseases, infection site, disease type, disease severity, biochemical findings, and 28-day mortality. We further stratified patients in the SAE group based on whether or not they survived for 28 days, and we compared the above data between these groups.

**Results:**

Of the 222 elderly sepsis patients, 132 (59.46%) had SAE. SAE patients were found to be significantly older than non-SAE patients. Both age and blood sodium concentrations were found to be associated with SAE risk, while elderly sepsis patients without underlying chronic obstructive pulmonary disease (COPD) have a relatively higher risk of developing SAE. The SAE group also had a significantly higher rate of 28-day mortality, and sequential organ failure assessment (SOFA) scores were a risk factor associated with 28-day mortality.

**Conclusion:**

Among elderly sepsis patients, SAE risk increases with advancing age, higher blood sodium concentrations, and without underlying COPD. SAE incidence is associated with a poorer prognosis, and SOFA scores are independent predictors of increased mortality among elderly SAE patients.

## 1. Introduction

Sepsis, defined as life-threatening organ dysfunction caused by a dysregulated host response to infection, affects millions of people worldwide each year and is a leading cause of global health loss [1, 2]. Over 70% of sepsis patients suffer from sepsis-associated encephalopathy (SAE) [3], resulting in severe symptoms including confusion, delirium, severe cognitive impairment, or potentially coma [4]. SAE may manifest before other sepsis symptoms [5]. Significantly, SAE can be associated with a dramatically poorer patient prognosis [6–8], and SAE has been considered the ultimate and critical cause of death for prolonged hospitalization [9]. Surviving SAE patients are likely to suffer from prolonged or permanent side effects, including anxiety, depression, dementia, reduced health-related quality of life, or suicidal behavior [10]. Despite its severity, the pathophysiological basis of SAE remains incompletely understood [11]. It is thought to be attributable, at least in part, to mechanisms including excessive microglial activation, impaired endothelial barrier function, and blood-brain barrier (BBB) dysfunction [4]. Given the relatively limited understanding of this condition, it is vital that further studies exploring the risk factors associated with SAE incidence and patient death be conducted.

Average population age values are rapidly rising in more developed nations such that elderly individuals account for >20% of the total population in some areas at present, and the majority of people outside of the Middle East and Africa will be 65 years of age or older by 2050 [12]. Advanced age is associated with an increased risk of sepsis [13] and higher mortality rates among those suffering from sepsis [14]. Elderly people >65 years old account for ~60% of all sepsis cases and ~80% of all sepsis-associated death [15], suggesting that SAE is also likely to manifest more often among individuals of more advanced age. Consistent with this possibility, SAE is not frequently observed among younger individuals despite being detected more frequently in the general population [16]. There is thus a need for further study of SAE incidence among the elderly.

Few studies have described the epidemiological basis for or risk factors associated with SAE incidence among the elderly. Previous studies have demonstrated a high incidence of SAE, which was a risk factor for poor prognosis [17], even when different sepsis diagnostic criteria are applied (sepsis 1.0 or sepsis 3.0) [18]. The SOFA scores were independent risk factors for predicting the occurrence and adverse outcome of SAE [17]. In addition to the severity of the condition, serum sodium was related to the presence of SAE [7, 8]. Delirium is one of the SAE manifestations, and age ≥ 65 years is a risk factor for sepsis-associated delirium (SAD) [19]. The risk factors for SAD increase as the severity of the condition for patients with sepsis increases [19]. Given the condition mentioned above, we hypothesized that the incidence of SAE is high among elderly sepsis patients, and there is a correlation between serum sodium and the occurrence of SAE, with SOFA scores being independent predictors of increased mortality among elderly SAE patients. To test this hypothesis, this retrospective study was designed to explore differences in clinical findings between elderly sepsis patients with and without SAE to understand better the etiology and risk factors of this disease among older adults.

## 2. Materials and methods

This study was conducted by the ethics committee of Hangzhou First People’s Hospital. Related patient information has been treated anonymously to protect patient privacy. We waived the requirement for informed consent because of the study’s retrospective nature. We followed our team’s primary methods of previous related research [17].

### 2.1. Patient selection

This retrospective analysis of all elderly people diagnosed with sepsis and admitted to our ICU between January 2017 and January 2022. Patient inclusion criteria were as follows: (1) Patients were diagnosed with sepsis based upon the sepsis 3.0 definition, which refers to the life-threatening multiple organ dysfunction caused by infection, and the multiple organ dysfunction was determined by an increase of at least 2 points in the Sequential Organ Failure Assessment score (SOFA score). (2) Patients were ≥60 years old, consistent with the World Health Organization definition of elderly age in developing nations. (3) Identification of SAE: Diagnosis of SAE is primarily based upon clinical presentation presently [20]. Concerning previous studies [8, 21], SAE was defined at ICU admission as a Glasgow coma scale (GCS) score of <15 or manifestations of delirium (including decreased psychomotor activity, disorientation, inattention, altered thinking, and agitation) confirmed by the Confusion Assessment Method for the Intensive Care Unit (CAM-ICU) in this article [8, 21]. GCS scores and CAM-ICU were evaluated on the 1st day of sepsis. We used GCS scores and CAM-ICU measured after temporary sedative/relaxant medication withdrawal for sedated patients at ICU admission. Patients admitted to the ICU due to acute brain dysfunction were excluded (e.g., encephalitis, meningitis, acute cerebral stroke, status epilepticus, hypertensive encephalopathy, metabolic encephalopathy, toxicosis, or traumatic brain injury). We identified 222 sepsis patients meeting these criteria, and they were separated into SAE and non-SAE groups.

### 2.2. Data collection

For each of the 222 patients included in this study, we collected the following data within 24 h of ICU admission: general clinical findings, Acute Physiology and Chronic Health Evaluation II (APACHE II) score, SOFA score, site of infection, hematological findings (white blood cell (WBC) count, platelet count (PLT), hematocrit (HCT), biochemical findings (serum sodium (Na), procalcitonin (PCT), and serum creatinine (Cr) levels), etiological information, and outcome indicators (days of hospitalization, 28-day mortality). Telephone-based follow-up was conducted after 28 days, and patient survival at this time point was used to stratify patients for analyses of survival-related risk factors.

### 2.3. Statistical analysis

All data were analyzed using SPSS 22.0 (SPSS Inc, NY, USA). Categorical and continuous variables are given as numbers (percentages) and medians [25th–75th percentiles], respectively, and were compared via the Mann–Whitney U tests, χ2 tests, or the Fisher’s exact tests, as appropriate. SAE-associated risk factors were identified via multivariate logistic regression analysis. Kaplan–Meier curves were analyzed with the log-rank test. p < 0.05 was the significance threshold.

## 3. Results

### 3.1. Baseline characteristics

**Figure 1:**
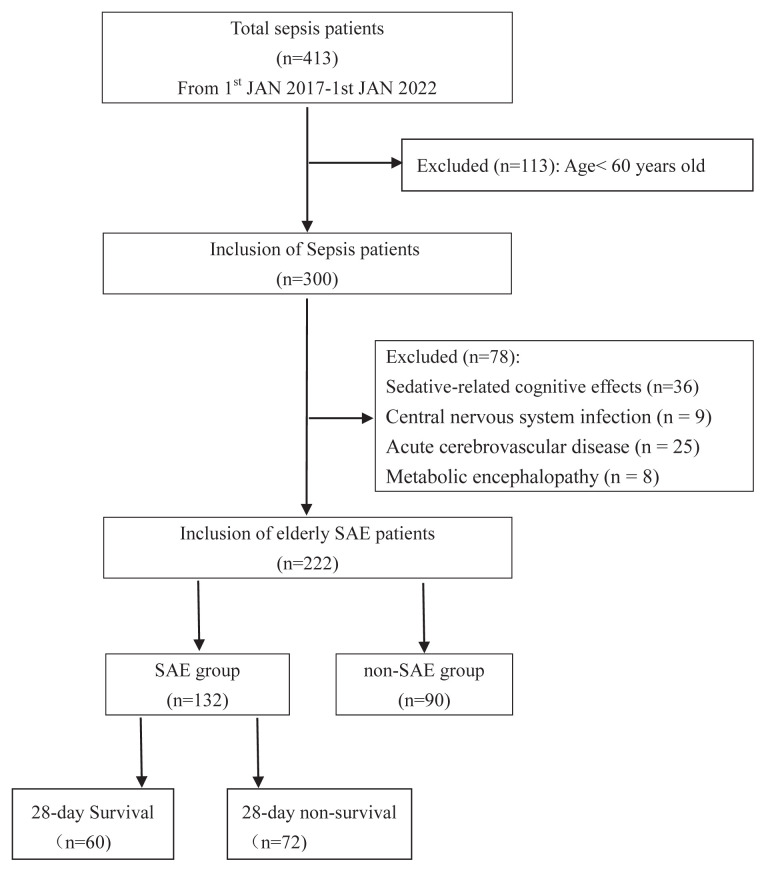
Flowchart of enrolled study participants. SAE = sepsis-associated encephalopathy.

During the study period, a total of 191 patients with sepsis were excluded from this study based on the inclusion mentioned above and exclusion criteria, yielding a cohort of 222 elderly sepsis patients admitted to our ICU (Figure 1). Of these patients, 132 (59.46%; 87 males) were diagnosed with SAE, and 90 (68 males) were not, and 192/222 (86.5%) were admitted to ICU due to medical disease. However, no significant differences were observed in disease types between SAE and non-SAE patients (p = 0.910). Among SAE individuals, 21/132 (15.91%) had delirium with a score on the GCS of 15. Coma was the most common manifestation of SAE in this study, and 29/132 (21.97%) had a score on the GCS of 13–14, 40/132 (30.3%) had a score on the GCS of 9–12, and 42/132 (31.82%) had a score on the GCS of 3–8. Additionally, seizures (n = 3/132, 2.27%) and neurological deficits (n = 1/132, 0.76%) were rarely observed. The median ages of the SAE and non-SAE groups were 77 [69, 83.75] and 73.5 [65, 81.0] years, respectively, with SAE patients being significantly older than non-SAE patients (p = 0.012). We also detected significant differences between these two groups for SOFA scores, APACHE II scores, and incidence of underlying diseases, including stroke, chronic obstructive pulmonary disease (COPD), and coronary heart disease (CHD) (All p < 0.05; Table 1).

### 3.2. Comparison of clinical and etiological findings between SAE and non-SAE patients

SAE patients had significantly higher infections in the respiratory tract (p = 0.028) and blood (p = 0.005) relative to non-SAE patients. In addition, these SAE patients had higher serum sodium concentrations (p = 0.006) and lower HCT values (p = 0.003) and platelet counts (p = 0.015) relative to their non-SAE counterparts. No significant differences were observed between groups for causative pathogens or infections of other tissues (p > 0.05; Table 2).

### 3.3. Comparison of primary outcomes between SAE and non-SAE patients

Duration of hospitalization and 28-day mortality was the primary outcomes monitored in our analyses. We observed no significant differences in hospitalization duration between the SAE and non-SAE groups. In contrast, however, we found that the 28-day mortality rate of SAE patients was significantly higher than that of non-SAE patients (54.5% vs. 28.9%, p < 0.001) (Table 2).

**Figure 2:**
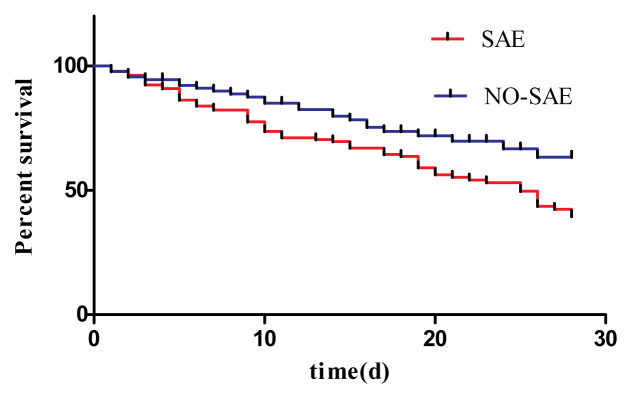
Kaplan–Meier analysis of the survival of sepsis patients over 28 days. The SAE and non-SAE patient groups were compared, revealing that the SAE group had a significantly high rate of 28-day mortality than the non-SAE group (HR = 1.720, 95% CI: 1.128–2.624, p = 0.012).

A Kaplan–Meier survival analysis further confirmed that SAE diagnosis was associated with significantly poorer 28-day survival among elderly sepsis patients (Figure 2; HR = 1.720, 95% CI: 1.128–2.624; p = 0.012).

### 3.4. Identification of SAE risk factors in the elderly

After adjusting for baseline characteristics, clinical manifestations and etiological findings, a subsequent multivariate analysis revealed that both age (OR = 1.054, 95% CI: 1.019–1.089, p = 0.002) and blood sodium concentration (OR per 1-mmol/L increment = 1.067, 95% CI: 1.023–1.113, p = 0.004) were independently associated with SAE risk among elderly sepsis patients, while elderly sepsis patients without a history of COPD (OR = 2.736, 95% CI: 1.338–5.597, p = 0.006) have a relatively higher risk of developing encephalopathy (Table 3).

### 3.5. Identification of mortality-related factors in elderly SAE patients

Lastly, we separated the 132 elderly SAE patients in our study cohort into two groups based on whether or not they were alive after 28 days (Table 4). The diagnosis of these patients includes severe pneumonia, abdominal infections, urinary tract infections, severe cholangitis, blood diseases, and skin soft-tissue infection. This yielded two populations: a group of nonsurvival patients (n = 72; 41 males; mean age = 77.68 ± 9.07 years) and a group of surviving patients (n = 60; 46 males; mean age = 75.67 ± 9.18 years). Overall mortality among SAE patients was 54.5%, with the males being higher in the nonsurvival group than the percentage of the females (56.9% vs. 43.1%; p = 0.017). We found that PCT level was significantly higher among nonsurviving patients (4.31 [1.6, 17.01] vs. 2.34 [0.51, 11.32], p = 0.049), as were mean SOFA scores (10.72 ± 3.82 vs. 8.33 ± 4.12, p < 0.001), whereas the percentage of patients with underlying COPD was significantly lower than that among surviving patients (13.9% vs. 20%; p = 0.008). No other significant differences in measured parameters were observed between these groups (p > 0.05) (Table 4). After adjusting for potential confounders, a multivariate analysis revealed that only SOFA scores were independently associated with 28-day mortality in elderly SAE patients (OR = 1.185, 95% CI: 1.074–1.307, p = 0.001) (Table 5).

## 4. Discussion

Our retrospective study indicates that over half of elderly sepsis patients suffer from SAE and were found to be significantly older than non-SAE patients. In this study, we found that blood sodium concentrations were associated with SAE risk independently, while elderly sepsis patients without underlying chronic obstructive pulmonary disease (COPD) have a relatively higher risk of developing SAE. This study confirms our hypothesis that SAE incidence is associated with a poorer prognosis, and SOFA scores are independent predictors of increased mortality among elderly SAE patients.

Sepsis patients admitted to our ICU were divided into three groups: those with internal disease, those undergoing emergency surgery, and those undergoing elective surgery. Consistent with Zhang et al. [7], we found that elderly sepsis patients primarily suffered from internal disease, with no significant SAE-related differences in disease types. However, a previous sizeable multicenter study found that SAE was more likely to occur in sepsis patients suffering from internal diseases [8]. Our failure to resolve any significant differences concerning disease etiology in the present study may be attributable to our relatively small sample size. Therefore, future large-scale studies or metaanalyses are warranted to explore better how SAE incidence is related to disease type.

BBB dysfunction can arise in older adults as a result of many conditions, including hypertension [22], epilepsy [23], and stroke [24]. We assessed the incidence of these underlying conditions in elderly sepsis patients, revealing that SAE patients were more likely to have suffered from CHD and stroke than were non-SAE patients. However, whether this relationship is causal remains to be further studied. There were no significant differences concerning SAE incidence in elderly individuals due to underlying hypertension, diabetes, arrhythmia, chronic liver disease, chronic kidney disease, or malignant tumors. Interestingly, in our study, elderly SAE patients had a lower frequency of underlying COPD than those without SAE. Prior studies have not detected a correlation between basal complicated COPD and SAE incidence [7, 8]. COPD is believed to be associated with hypercapnia, and Albayrak et al. found that total cerebral blood flow volumes were significantly higher in COPD patients and hypercapnic ones [25]. Thus, we guess that the increase in cerebral blood flow in COPD patients might improve cerebral perfusion and lead to a lower risk of SAE. Further prospective studies will, therefore, be needed to disentangle better the relationship between COPD, arterial blood carbon dioxide partial pressure, and SAE. In a univariate analysis, we also found that SAE incidence was higher among elderly sepsis patients with higher SOFA and APACHE II scores. Note that GCS scores of < 15 were used to diagnose patients with SAE; however, GCS scores are a component of SOFA and APACHE II scores. Patients with higher SOFA and APACHE II scores are more likely to have SAE, potentially biasing this conclusion.

Recent multicenter studies have suggested that *Staphylococcus aureus* infection is associated with higher SAE incidence, whereas the source or spread of the infection is not [8]. However, our study did not detect any relationship between pathogen type and SAE incidence. Instead, we found that SAE incidence was associated with blood or respiratory tract infections. Respiratory tract infections are the most common source of infection in patients with sepsis, including the elderly population. Specifically, 33% of sepsis cases were due to respiratory tract infections, and 32% to genitourinary infections [26]. Few studies have explored the relationship between respiratory tract infections and SAE. As one of the SAE manifestations, delirium symptoms independently predict 1-year mortality in patients with severe pneumonia [27]. Interestingly, delirium may also represent the phenomenological symptom of inadequate oxygenation of vital tissues [28], while respiratory tract infections may impact oxygen delivery, considering that oxygenation is one of the primary roles of the respiratory system. Metabolic disorders have also been related to SAE risk [8]. We found higher serum sodium, lower PLT, and lower HCT values associated with increased SAE risk in univariate analysis, with only elevated serum sodium levels remaining associated with SAE incidence independently in subsequent multivariate analysis. Hypernatremia is linked to multiple neurological manifestations, including encephalopathy, seizures, delirium, and related alterations in consciousness [29, 30]. Hypernatremia is also independently associated with delirium and mortality among patients admitted to the ICU [31]. We could not further explore this potential relationship between sodium levels and SAE incidence in our elderly population. We also found that SAE incidence was not associated with an increase in hospitalization duration, although it was associated with significantly reduced 28-day survival, with higher SOFA scores being independently associated with the risk of mortality. We speculated that increased disease severity is associated with poorer patient outcomes.

Given the steadily increasing average age of many nations, our study offers a clear advantage by focusing specifically on SAE incidence among elderly sepsis patients. Few studies to date have explored the epidemiology of SAE in elderly populations to understand the risk factors underlying the incidence of this condition. Our study offers new data in support of these previous studies. However, there are several limitations to the present analysis. For one, this was a retrospective case-control study. Therefore, future prospective studies will be required to establish any causal relationships between the risk factors identified herein and SAE incidence among elderly sepsis patients. The limited sample size of this study also constrains our statistical power, potentially leading to inaccurate conclusions in some contexts. Furthermore, there are no widely accepted diagnostic criteria for SAE, and the criteria used in the present study were subjective diagnostic methods such as GCS scores, CAM-ICU, and defined exclusion criteria. Therefore, it will be essential to identify SAE based on other objective criteria in future analyses, including EEG and somatosensory evoked potentials. Our analyses also focused explicitly on parameters of interest in sepsis patients at the time of diagnosis, and as such, they do not offer any insight into the dynamics of these parameters over time since they pertain to SAE risk.

In summary, this study showed that SAE occurred at higher rates among elderly patients of more advanced age. SAE was also more likely to affect elderly sepsis patients with higher serum sodium levels, while higher SOFA scores, higher APACHE II scores, lower PLT, lower HCT, underlying CHD, underlying stroke, and infections of the blood or respiratory tract were all identified as possible SAE-associated risk factors. In turn, elderly sepsis patients with underlying COPD have a lower risk of developing SAE. Elderly sepsis patients affected by SAE were found to have a poorer prognosis than patients not affected by this condition, with higher SOFA scores being independently associated with mortality risk. Future studies will be required to fully validate the prognostic value of the indicators studied in the present article, extending our findings to explore dynamic changes in SAE morbidity and mortality among elderly populations.

## Figures and Tables

**Table 1:** Patients’ baseline characteristics.

Variable	All patients n = 222	SAE n = 132	Non-SAE n = 90	p-value
Age, years	76[68, 82]	77[69,83.75]	73.5[65, 81.0]	0.012
Sex				
male	155 (69.8)	87 (65.9)	68 (75.6)	0.124
female	67 (30.2)	45 (34.1)	22 (24.4)	
Disease type				
Medical disease,	192 (86.5)	115 (87.1)	77 (85.6)	0.910
Emergency surgery	17 (7.7)	10 (7.6)	7 (7.8)	
Elective surgery	13 (5.9)	7 (5.3)	6 (6.7)	
Underlying diseases				
Hypertension	128 (57.7)	76 (57.6)	52 (57.8)	0.976
Diabetes	60 (27.0)	38 (28.8)	22 (24.4)	0.474
Coronary heart disease	61 (27.5)	44 (33.3)	17 (18.9)	0.018
Arrhythmia	44 (19.8)	31 (23.5)	13 (14.4)	0.097
COPD	47 (21.2)	22 (16.7)	25 (27.8)	0.047
Chronic liver disease	12 (5.4)	6 (4.5)	6 (6.7)	0.553*
Chronic kidney disease	13 (5.9)	6 (4.5)	7 (7.8)	0.314
Malignant tumor	53 (23.9)	31 (23.5)	22 (24.4)	0.869
Stroke	63 (28.4)	44 (33.3)	19 (21.1)	0.047
Disease severity				
SOFA	7 [4, 11]	10 [6, 13]	4 [2, 7]	<0.001**
APACHE II	17 [12, 27]	23 [15, 31]	12 [9, 17]	<0.001**

COPD: chronic obstructive pulmonary disease; SOFA: sequential organ failure assessment; APACHE II: Acute Physiology, Age and Chronic Health Evaluation II;

* Statistical analysis using Fisher’s exact probability method;

** Statistical analysis using the Mann–Whitney test. Chi-squared test was used for statistical treatment of p-values without “*” or “**” annotation.

**Table 2:** Comparison of clinical and etiological findings and primary outcomes in elderly sepsis patients.

Variable	All patients n = 222	SAE n = 132	Non-SAE n = 90	p-value
Infection source (%)				
Respiratory tract	163 (73.4)	104 (78.8)	59 (65.6)	0.028
Gastrointestinal tract	12 (5.4)	10 (7.6)	2 (2.2)	0.129*
Biliary tract	24 (10.8)	12 (9.1)	12 (13.3)	0.318
Intraabdominal	34 (15.3)	18 (13.6)	16 (17.8)	0.400
Urinary tract	34 (15.3)	24 (18.2)	10 (11.1)	0.151
Bloodstream infection	19 (8.6)	17 (12.9)	2 (2.2)	0.005
Skin and soft tissue	10 (4.5)	4 (3.0)	6 (6.7)	0.200
Main microorganisms, n (%)				
*Staphylococcus*	35 (15.8)	22 (16.7)	13 (14.4)	0.656
*Enterococcus*	17 (7.7)	9 (6.82)	8 (8.9)	0.569
*Escherichia coli*	25 (11.3)	14 (10.6)	11 (12.2)	0.708
*Acinetobacter baumannii*	60 (27.0)	40 (30.3)	23 (25.6)	0.441
*Pseudomonas aeruginosa*	22 (9.9)	14 (10.6)	8 (8.9)	0.674
*Stenotrophomonas maltophilia*	14 (6.3)	9 (6.82)	5 (5.6)	0.704
*Klebsiella pneumoniae*	39 (17.6)	22 (16.7)	17 (18.9)	0.669
*Burkholderia cepacia*	10 (4.5)	5 (3.8)	5 (5.6)	0.531*
*Serratia marcescens*	5 (2.3)	4 (3.0)	1 (1.1)	0.651*
Fungus	43 (19.4)	27 (20.5)	16 (17.8)	0.620
Biochemical indicators				
WBC (*10^9^/L)	11.10[7.33, 15.83]	10.76[7.65, 5.32]	11.78 [7.05, 16.00]	0.922**
PCT (ng/mL)	3.43[1.01, 13.79]	3.72[0.99, 14.56]	2.89 [1.04, 12.96]	0.491**
PLT (*10^9^/L)	122 [59.5, 183]	100[52.25, 174]	151[76.5, 206]	0.015**
HCT (%)	29.11[24.5, 34.03]	29.11[22.8, 32.25]	30.95[26.58, 34.85]	0.003**
Na (mmol/L)	140[136, 145]	141[136.7, 146]	139.4[135, 141]	0.006**
Cr (umol/L)	84 [60.93, 135.2]	83.4 [60.1, 134.6]	85.5[68.25, 136.2]	0.719**
Primary outcomes				
Duration of hospitalization	19.73 [12.75, 25]	19.73 [10.25, 26]	21.5 [14, 23.54]	0.336**
Duration of hospitalization in survival patients	19.73 [12.75, 25]	19.73 [10.25, 26]	21.5 [14, 23.54]	0.336**
28-day mortality (%)	98 (44.1)	72 (54.5)	26 (28.9)	<0.001

WBC: white blood cell; PCT: procalcitonin; PLT: blood platelet; HCT: red blood cell specific volume; Na: serum sodium levels; Cr: Serum creatinine.

* Statistical analysis using Fisher’s exact probability method;

** Statistical analysis using the Mann–Whitney test. Chi-squared test was used for statistical treatment of p-values without “*” or “**” annotation.

**Table 3:** Subsequent multivariate analysis of SAE risk factors in the elderly.

Value	OR	95% CI	p-value
Age	1.054	1.019~1.089	0.002
Without a history of COPD	2.736	1.338~5.597	0.006
Na (per 1-mmol/L increment)	1.067	1.023~1.113	0.004

COPD: chronic obstructive pulmonary disease; Na: serum sodium levels.

**Table 4:** Baseline characteristics in SAE patients.

Variable	All patients n = 132	Nonsurvival n = 72	Survival n = 60	p-value
Age, years	77[69, 83.75]	77.68±9.07	75.67±9.18	0.209
Sex				
male	87(65.9)	41(56.9)	46(76.7)	0.017
female	45(34.1)	31(43.1)	14(23.3)	
Disease type				
Medical disease,	115(87.1)	64(88.8)	51(85)	0.628
Emergency surgery	10(7.6)	4(5.6)	6(10)	
Elective surgery	7(5.3)	4(5.6)	3(5)	
Underlying diseases				
Hypertension	76(57.6)	43(59.7)	33(55)	0.585
Diabetes	38(28.8)	23(31.9)	15(25)	0.380
Coronary heart disease	44(33.3)	23(31.9)	21(35)	0.711
Arrhythmia	31(23.5)	17(23.6)	14(23.3)	0.970
COPD	22(16.7)	10(13.9)	12(20)	0.008
Chronic liver disease	6(4.5)	3(4.2)	3(5)	1.000*
Chronic kidney disease	6(4.5)	4(5.6)	2(3.3)	0.688*
Malignant tumor	31(23.5)	14(19.4)	17(28.3)	0.230
Stroke	44(33.3)	22(30.6)	22(36.7)	0.458
Disease severity				
SOFA	10 [6, 13]	10.72±3.82	8.33±4.12	<0.001
APACHE II	23 [15, 31]	24 [18.25, 30]	19[13, 31]	0.077**
Infection source (%)				
Respiratory tract	104(78.8)	60(83.3)	44(73.3)	0.162
Gastrointestinal tract	10(7.6)	4(5.6)	6(10)	0.511*
Biliary tract	12(9.1)	9(12.5)	3(5)	0.136
Intraabdominal	18(13.6)	9(12.5)	9(15)	0.677
Urinary tract	24(18.2)	12(16.7)	12(20)	0.621
Bloodstream infection	17(12.9)	7(9.7)	10(16.7)	0.236
Skin and soft tissue	4(3.0)	1(1.4)	3(5)	0.329*
Main microorganisms, n (%)				
*Staphylococcus*	22(16.7)	16(22.2)	6(10)	0.061
*Enterococcus*	9(6.82)	7(9.7)	2(3.3)	0.181*
*Escherichia coli*	14(10.6)	8(11.1)	6(10)	0.837
*Acinetobacter baumannii*	40(30.3)	26(36.1)	14(23.3)	0.112
*Pseudomonas aeruginosa*	14(10.6)	9(12.5)	5(8.3)	0.439
*Stenotrophomonas maltophilia*	9(6.82)	4(5.6)	5(8.3)	1.000*
*Klebsiella pneumoniae*	22(16.7)	10(13.9)	12(20)	0.348
*Burkholderia cepacia*	5(3.8)	3(4.2)	2(3.3)	1.000*
*Serratia marcescens*	4(3.0)	1(1.4)	3(5)	0.329*
Fungus	27(20.5)	11(15.3)	16(26.7)	0.106
Biochemical indicators				
WBC (*10^9^/L)	10.76[7.65, 5.32]	11.1[8.85, 17.47]	9.95[6.92, 14.91]	0.081**
PCT (ng/mL)	3.72[0.99, 14.56]	4.31[1.6, 17.01]	2.34[0.51, 11.32]	0.049**
PLT (*10^9^/L)	100[52.25, 174]	96[49.25, 163.8]	131[56.5, 190]	0.222**
HCT (%)	29.11[22.8, 32.25]	29.35[22.73, 40.28]	25.55[23.15, 31.78]	0.066**
Na (mmol/L)	141[136.7, 146]	142[137.4, 145.1]	140.1[136.3, 147.7]	0.998**
Cr (umol/L)	83.4 [60.1, 134.6]	83.9[60.18, 124.4]	81.5 [60.1, 150]	0.798**

COPD: chronic obstructive pulmonary disease; SOFA: sequential organ failure assessment; APACHE II: Acute Physiology, Age and Chronic Health Evaluation II; WBC: white blood cell; PCT: procalcitonin; PLT: blood platelet; HCT: red blood cell specific volume; Na: serum sodium levels; Cr: Serum creatinine.

* Statistical analysis using Fisher’s exact probability method;

** Statistical analysis using the Mann–Whitney test. Chi-squared test was used for statistical treatment of p-values without “*” or “**” annotation.

**Table 5:** Multivariate analysis of 28-day mortality in elderly SAE patients.

Value	OR	95% CI	p-value
SOFA	1.185	1.074~1.307	0.001

SOFA: sequential organ failure assessment.
